# TIPE2 Negatively Regulates Inflammation by Switching Arginine Metabolism from Nitric Oxide Synthase to Arginase

**DOI:** 10.1371/journal.pone.0096508

**Published:** 2014-05-07

**Authors:** Yunwei Lou, Guizhong Zhang, Minghong Geng, Wenqian Zhang, Jian Cui, Suxia Liu

**Affiliations:** Department of Immunology, Shandong University School of Medicine, Ji'nan, P.R. China; Vanderbilt University School of Medicine, United States of America

## Abstract

TIPE2, the tumor necrosis factor (TNF)-alpha-induced protein 8-like 2 (TNFAIP8L2), plays an essential role in maintaining immune homeostasis. It is highly expressed in macrophages and negatively regulates inflammation through inhibiting Toll-like receptor signaling. In this paper, we utilized RAW264.7 cells stably transfected with a TIPE2 expression plasmid, as well as TIPE2-deficient macrophages to study the roles of TIPE2 in LPS-induced nitric oxide (NO) and urea production. The results showed that TIPE2-deficiency significantly upregulated the levels of iNOS expression and NO production in LPS-stimulated macrophages, but decreased mRNA levels of arginase I and urea production. However, TIPE2 overexpression in macrophages was capable of downregulating protein levels of LPS-induced iNOS and NO, but generated greater levels of arginase I and urea production. Furthermore, *TIPE2^−/−^* mice had higher iNOS protein levels in lung and liver and higher plasma NO concentrations, but lower levels of liver arginase I compared to LPS-treated WT controls. Interestingly, significant increases in IκB degradation and phosphorylation of JNK, p38, and IκB were observed in TIPE2-deficient macrophages following LPS challenge. These results strongly suggest that TIPE2 plays an important role in shifting L-arginase metabolism from production of NO to urea, during host inflammatory response.

## Introduction

TNFAIP8L2, the tumor necrosis factor (TNF)-alpha-induced protein 8-like 2 (also known as TIPE2), is a new member of the TNFAIP8 (also called SCC-S2, GG2-1, and MDC-3.13) family [1]–[4]. TIPE2 plays an essential role in the maintenance of immune homeostasis by interfering with T cell receptor (TCR) and Toll-like receptor (TLR) signaling pathways [1], [5]–[6]. Recently, studies have focused on the TIPE2 protein because it is considered to be a negative regulator not only in inflammation but also in carcinogenesis [1], [5]–[7]. TIPE2 deficiency in mice causes fetal inflammatory diseases [1] and its abnormal expression in humans is associated with infectious diseases, diabetic nephropathy, stroke and atherosclerosis [8]–[12].

L-arginine (L-arg) is the substrate for both nitric oxide synthase (NOS) and arginase. NOS uses L-arg as a substrate in the synthesis of L-citrulline and NO, while arginase catalyzes the conversion of L-arg to produce L-ornithine and urea. There are two described isoforms of arginase [13]. arginase I (Arg 1) has been referred to as the hepatic isoform, its expression can be induced by lipopolysaccharide (LPS) and alterations in oxygen tension in a wide variety of cells and tissues [14]–[16]. arginase I I(Arg 2) has been described as an extra-hepatic isoform and is induced by LPS, IFN-γ, and hyperoxia [13]–[14], [16]. The L-ornithine produced by arginase is vital to tissue repair processes following injury and is considered to be involved in healing [17]–[18].

There are three described isoforms of NOS, neuronal NOS (nNOS), endothelial NOS (eNOS), and induced nitric oxide synthase (iNOS). The maintenance of a constitutive but limited supply of NO via eNOS is crucial for maintaining vascular health, while the NO produced by iNOS has a wide variety of physiological functions in inflammation [19]–[21]. It is abundantly expressed in macrophages [22] and contributes to tissue damage at sites of inflammation, such as atherosclerotic lesions [23]–[24]. Recently, studies showed that the deletion of arginase II could increase iNOS protein levels and NO generation by causing intracellular depletion of L-arginine in reponse to infection by H. pylori [1], [12], [25]–[26]. Thus the idea that NOS and arginase may have important yet divergent roles in the immune response has lead us to study the mechanisms that allow macrophages to redirect L-arg metabolism from NOS to arginase. Early studies show that TIPE2 is highly expressed in macrophages and can negatively regulate inflammation through inhibiting NF-κB, JNK, and p38 pathways [1], [12], [25]–[26]. It has been reported that the mitogen-activated protein kinases (MAPK) and NF-κB pathways contribute to iNOS induction in LPS-stimulated RAW264.7 cells [27]–[28]. Thus we hypothesize that TIPE2 negatively regulates inflammation by switching arginine metabolism from LPS-induced iNOS to arginase in macrophages, resulting in changing L-arg metabolism from the production of NO and L-citrulline to the production of urea and L-ornithine. To test this hypothesis, we utilized RAW264.7 cells stably transfected with a TIPE2 expression vector, as well as thioglycollate-elicited peritoneal macrophages from *TIPE2^−/−^* mice, to study the roles of TIPE2 in LPS-induced NO and urea production. Our results strongly suggest that TIPE2 plays an important role in shifting L-arg metabolism from production of NO to urea during host inflammatory response.

## Materials and Methods

### RAW264.7 culture

Murine macrophage cell line Raw264.7 was obtained from the American Type Culture Collection (Manassas, VA, USA) and cultured in DMEM (GIBCO-BRL, Carlsbad, CA, USA) supplemented with 10% fetal bovine serum (Gibco-BRL, Carlsbad, CA, USA) at 37 °C in a humidified atmosphere containing 5% CO_2_. Cells were transfected with a TIPE2 expression vector (pRK5-TIPE2) or pRK5 alone using Lipofectamine 2000 (Invitrogen, Carlsbad, CA, USA) according to the manufacturer's protocol. The cells were then selected in medium with 500 µg/mL G418 (Invitrogen) for two weeks, then the resistant clones were isolated, expanded, and used for the following experiments.

### Experimental Animals

The male TIPE2 knockout (*TIPE2^−/−^*) mice in C57BL/6J background (8 to 10 weeks old) have previously been described [1]. Male Wild type (WT) mice in C57BL/6J background were purchased from Shanghai Laboratory Animal Center of Chinese Academy of Science (Shanghai, China), and were 8 to 10 weeks old at the time of entry into the study. All mice were housed in the Animal Facilities of Shandong University under pathogen-free conditions throughout the experiments. All experiments with animals were performed according to the guidelines of the Animal Management Rules of the Chinese Ministry of Health (document No. 55, 2001) and were approved by the Animal Ethical Committee of Shandong University.

### Peritoneal Macrophage Isolation and Culture

For isolation of elicited peritoneal macrophages, age and sex-matched WT and TIPE2-deficient mice were injected intraperitoneally with 1.0 mL of 3% sterile thioglycollate broth (Sigma-Aldrich). Four days after injection, cells were harvested by intraperitoneal lavage with 10 mL ice-cold PBS and were extensively washed using ice-cold PBS. Cells were then seeded in DMEM medium (GIBCO-BRL, Carlsbad, CA, USA) with 10% FBS, 100 U/mL penicillin, and 100 µg/mL streptomycin for 4 hours and adherent cells were taken as peritoneal macrophages.

### Treatment of Animals

WT or *TIPE2^−/−^* mice were treated with 1.5 mg/kg LPS (Sigma-Aldrich, St. Louis, MO, USA) or PBS intraperitoneal administration. At 0 h, 3 h, and 24 h after treatment, mice were euthanized for blood sampling, and then the lung and liver tissues were collected and stored at −80°C until use.

### Analysis of NO

Primary peritoneal macrophages or RAW264.7 cells incubated with DMEM medium containing 10% FBS overnight before stimulation were plated at 3×10^5^ cell/well in 24-well culture plates (Corning). After the cells were treated with 100 ng/mL LPS (Sigma-Aldrich) for 24 h, culture medium was collected. Blood from WT and *TIPE2^−/−^* mice was collected by cardiac puncture after being euthanized for preparation of sera. Nitrite levels in culture medium or in sera were determined by the Griess assay [29]. The cells were collected to determine the expression levels of mRNA or protein of iNOS by the methods of q-PCR or Western blotting, respectively. The experiments were performed in triplicate.

### Urea assay

The samples were collected as described above. Urea levels in culture medium or in sera were determined with the use of standard enzymatic methods and commercial kits (Roche Diagnostics, Indianapolis, IN), according to the manufacturer's guidelines.

### Quantitative real-time PCR

Total RNA of cells or tissues was prepared using Trizol reagent (Invitrogen). Reverse transcription and quantitative PCR (qPCR) of interested genes was performed as previously described [8]. Amplification conditions were: 95 °C for 3 min and then 95 °C for 15 s, 55 °C for 15 s and 72 °C for 30 s for 40 cycles. Primers used for this study were synthesized by Invitrogen Corporation and shown as follows: 5′-TCAGAAACATCCAAGGCCAGAC-3′ (sense) and 5′-CGGACCGACCAGCCATTTTAC-3′ (antisense) for TIPE2; 5′-CACCTTGGAGTTCACCCAGT-3′ (sense) and 5′-ACCACTCGTACTTGGGATGC-3′ (antisense) for iNOS; 5′-AAGAAAAGGCCGATTCACCT-3′ (sense) and 5′-CACCTCCTCTGCTGTCTTCC-3′ (antisense) for Arg I; 5′-GGATCCAGAAGGTGATGGAA-3′ (sense) and 5′-AGAGCTGACAGCAACCCTGT-3′ (antisense) for Arg II; 5′-TGCGTGACATCAAAGAGAAG-3′ (sense) and 5′-TCCATACCCAAGAAGGAAGG-3′ (antisense) for β-actin.

### Western blotting

Western blotting was performed as previously described [8]. The following primary antibodies were used: anti-iNOS (1∶1000), anti-ERK and p-ERK (1∶1000), anti-p38 and p-p38 (1∶1000), anti-JNK and p-JNK (1∶500), anti-IκB and p-IκB (1∶500), anti-β-actin (1∶1000). All antibodies were purchased from Cell Signaling Technology (Beverly, MA, USA). Rabbit anti-mouse TIPE2 polyclonal antibody was used as previously described [8]. After incubating with primary antibodies, the membranes were incubated with goat anti-rabbit Ig G or goat anti-mouse Ig G conjugated with peroxidase. After washing, bound peroxidase activity was detected by the ECL detection system (ECL, F-cheiBIsi.6pro, DNR, Israel) using the SuperSignal West Pico trial kit (Pierce Biotechnology, Rockford, IL, USA).

### Statistical analysis

Results were expressed as mean ±SE. An unpaired t-test was used to determine the significance of differences between groups. Levels of significance for comparisons between multiple groups were determined by one-way ANOVA. A value of *P*<0.05 was considered statistically significant. All analyses were performed using the Prism 5.0 for Windows (Graphpad Software, San Diego, Calif.).

## Results

### TIPE2 overexpression attenuates LPS-induced iNOS expression in RAW264.7 cells

**Figure 1:**
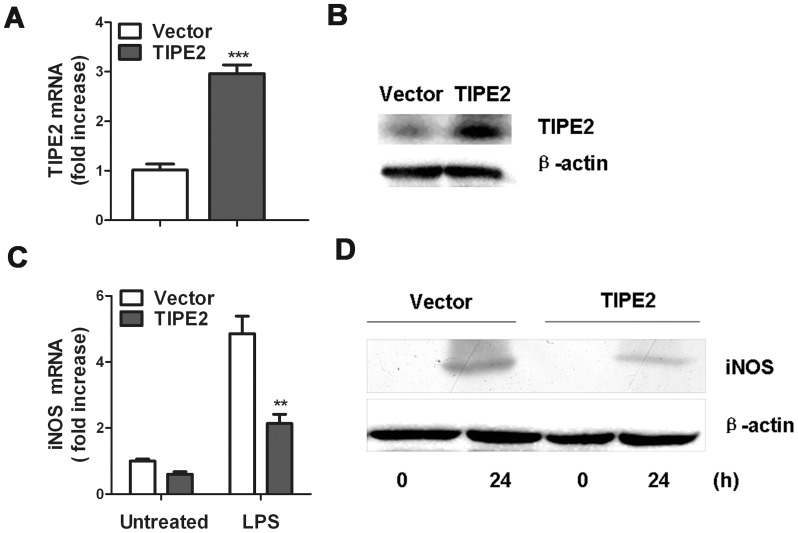
Effect of TIPE2 Overexpression on LPS-induced iNOS expression. RAW264.7 cells were stably transfected with TIPE2 plasmid or vector control. TIPE2 expression levels were determined by quantitative RT-PCR (**A**) and Western blot (**B**), respectively. For quantitative PCR, the results were presented as folds expression of TIPE2 RNA to that of β-actin. TIPE2 overexpression RAW264.7 cells or control cells were treated with 100 ng/mL LPS for 24 h, and iNOS mRNA (**C**) and protein (**D**) levels were detected by quantitative PCR and Western blot, respectively. Data are shown as mean ±SE of one representative experiment. **P<0.01; ***P<0.001.

As shown in Figure 1A and 1B, expression of TIPE2, both at the mRNA and proteinlevels, was intensely detected in RAW264.7 cells stable transfected with TIPE2 plasmid. Low level of iNOS mRNA was detected in RAW264.7 cells without treatment (Figure 1C, left lane). Upon LPS stimulation, iNOS mRNA levels were dramatically increased in both TIPE2-overexpressing RAW264.7 macrophages and the control cells (Figure 1C, right lane). However, the iNOS mRNA levels in TIPE2 overexpression cells treated with LPS were much lower than LPS-treated controls, suggesting that enhanced TIPE2 expression inhibited the induction of iNOS mRNA by LPS challenge. Furthermore, LPS treatment resulted in substantially greater levels of iNOS protein in RAW264.7 cells (Figure 1D). However, compared with LPS-treated cells transfected with empty vector control, the levels of iNOS protein in TIPE2 overexpression cells treated with LPS were much lower. These data further confirm that TIPE2 can attenuate the expression of iNOS in RAW264.7 cells.

### TIPE2 overexpression increases LPS-induced arginase I expression but not arginase II

**Figure 2:**
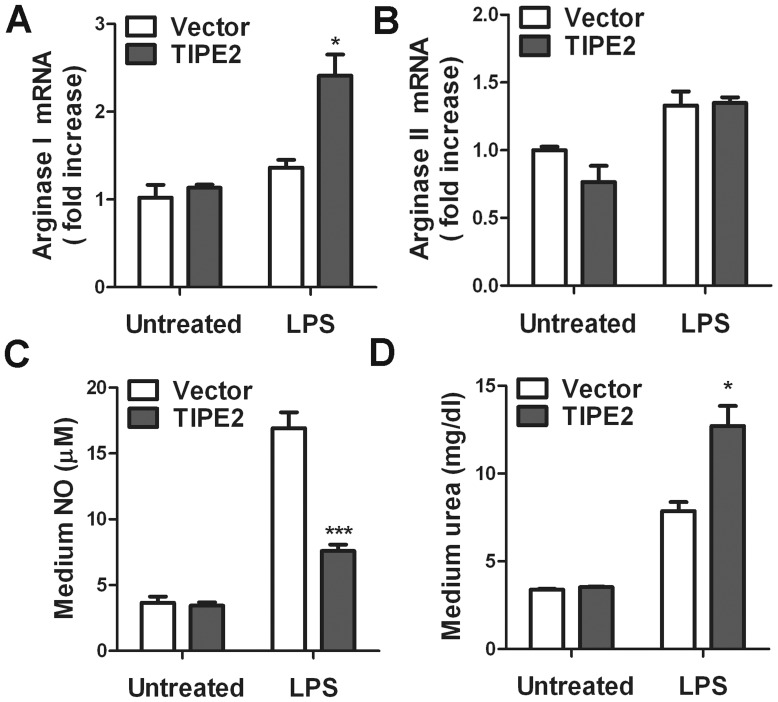
Effect of TIPE2 overexpression on LPS-induced arginases mRNA expression, NO and urea production in macrophages. A and B, RAW264.7 cells overexpressing TIPE2 or control cells were treated with 100/mL LPS for 4 h, and expression levels of arginaseI and arginase II mRNA were determined by quantitative PCR. Culture media were collected for NO and urea measurement (C and D). Data are shown as means ±SE of triplicates from an experiment that was repeated a total of three times with similar results. *P<0.05.

As shown in Figure 2A, there is no significant difference in arginasImRNA levels between TIPE2 overexpression cells and empty vector controls without LPS-treatment, indicating that exogenous TIPE2 has little effect on arginase I expression. However, upon LPS treatment, the mRNA levels of arginase I were increased significantly in cells overexpressing TIPE2 compared to vector controls (*P*<0.05). As shown in Figure 2B, the mRNA levels of arginasII were increased in both cells overexpressing TIPE2 and vector controls on LPS treatment. However, there was no significant difference in the induction of arginase I in RAW264.7 cells treated with LPS regardless of whether or not TIPE2 was overexpressed. These results suggest that TIPE2 overexpression increases LPS-induced arginase I production but has little effect on arginase II expression.

### TIPE2 overexpression decreases LPS-induced NO production but increases urea levels in RAW264.7 cells

LPS-treatment resulted in increased NO production in both TIPE2 overexpression cells and vector controls (Figure 2C). However, the levels of NO in TIPE2 overexpression cells were much lower than vector controls, indicating that TIPE2 overexpression attenuated LPS-induced NO production. As shown in Figure 2D, LPS stimulation resulted in markedly increased urea production in both TIPE2 overexpression cells and controls. Interestingly, the levels of urea were much higher than vector controls. There was a significant difference between these two kinds of cells. These data suggest that TIPE2 overexpression attenuates LPS-induced NO production but increases urea levels in RAW264.7 macrophages.

### TIPE2-deficiency increases LPS-induced NO production but decreases urea production in primary macrophages

**Figure 3:**
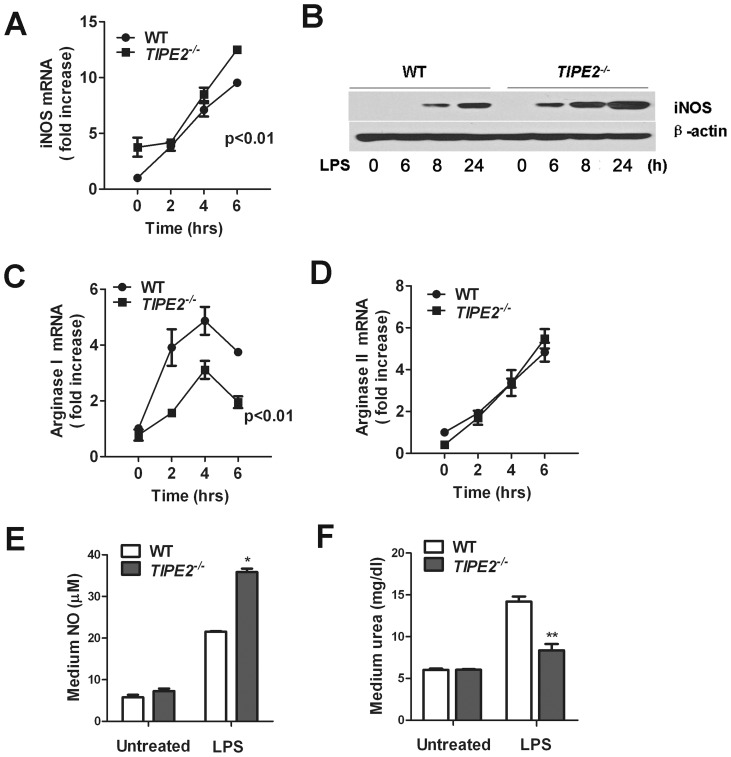
TIPE2 deficiency increases NO production but decreases urea production in macropahges. Peritoneal macrophages from WT and *TIPE2^−/−^* mice were treated with 100 ng/mL LPS for 0 h, 3 h, and 24 h. iNOS mRNA (**A**) and protein (**B**) levels were determined by quantitative PCR and Western blot, respectively. Expression levels of arginase I and arginase II mRNA were examined by quantitative RT-PCR (**D** and **C**). Cells were stimulated with 100 ng/mL LPS for 24 h, and culture supernatants were harvested for measurement of NO and urea (**E** and **F**). Data are shown as means ±SE (n = 4) of one representative experiment. *P<0.05.

As shown in Figure 3A, after treatment with LPS, the levels of iNOS mRNA in TIPE2-deficient macrophages from *TIPE2^−/−^* mice were significantly higher than macrophages from WT controls. As a consequence, the levels of iNOS protein at 24 h treated with LPS in TIPE2-deficient macrophages were significantly increased compared to WT controls (Figure 3B). LPS-treatment resulted in a marked increase in the arginase I mRNA level in both TIPE2-deficient macrophages and WT controls (Figure 3C). However, the levels of arginase I mRNA in TIPE2-deficient macrophages were much lower than WT cells, indicating that TIPE2 deficiency attenuated LPS-induced arginase I transcription. As shown in Figure 3D, TIPE2 deficiency had little effect on arginasII production.

We also detected the NO levels in the supernatant from TIPE2-deficient macrophages and WT control cells. As shown in Figure 3E and 3F, TIPE2-deficient macrophages produced more NO than WT cells after LPS challenge, but less urea production in TIPE2-deficient macrophages than cells from WT mice, suggesting that in the absence of TIPE2, NO production was enhanced, whereas urea production was decreased.

### TIPE2-deficient mice exhibit increased iNOS induction and NO production following LPS challenge

**Figure 4:**
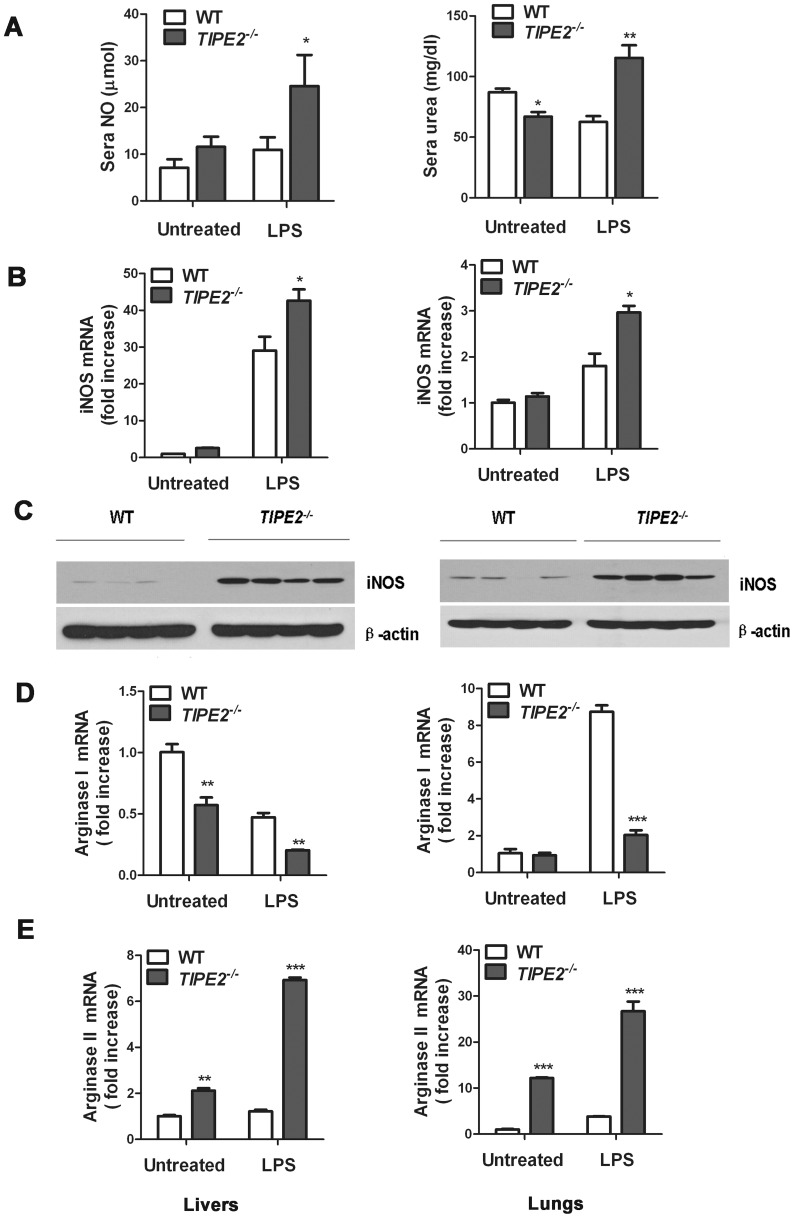
TIPE2-deficient deficient mice exhibit greater iNOS induction and NO production in response to LPS challenge compared to WT controls. WT and TIPE2^−/−^ mice injected intraperitoneal with phosphate buffered saline (PBS) or with LPS (1.5 mg/kg body weight) and sacrificed 3 or 24 h later. Sera concentration of NO and urea were examined (A and B). Liver and lung tissues of these animals were collected to extract total RNA and protein. The mRNA levels of iNOS, arginase I and arginase II in livers (B, D and E, left panels) and lungs (B, D and E, right panels) were examined by quantitative PCR at 3 h post-PBS or LPS challenge. iNOS protein levels in the livers (C, left panel) and lungs (C, right panel) were examined by Western blot at 24 h post-LPS challenge. Data are shown as means ±SE (n = 4) of one representative experiment. *P<0.05; **P<0.01; ***P<0.001.

As shown in Figure 4A, the concentrations of NO in sera in TIPE2-deficient mice were higher than those in WT controls following LPS challenge, while concentrations of urea in sera changed in an opposite manner. The concentrations of urea in WT mice were decreased at 24 h after LPS challenge, whereas the levels of urea were increased significantly in *TIPE2^−/−^* mice (Figure 4A). Additionally, we detected the expression levels of iNOS, arginasI, and arginasII in liver and lung tissues from *TIPE2^−/−^* mice and WT controls following LPS challenge, respectively. The levels of iNOS mRNA in both liver and lung from *TIPE2^−/−^* mice were increased compared to tissues from WT mice (Figure 4B), resulting in higher levels of iNOS protein in tissues from *TIPE2^−/−^* mice than WT controls (Figure 4C). The mRNA levels of arginasI were decreased in both liver and lung tissues from *TIPE2^−/−^* mice compared to tissues from WT following LPS challenge (Figure 4D), whereas the levels of arginaseII mRNA were increased markedly in both liver and lung from *TIPE2^−/−^* mice (Figure 4E), suggesting that these high expression levels of arginaseII in tissues might contribute to high levels of urea production in sera.

### LPS challenge increases IκB, JNK and p38 phosphorylation in TIPE2-deficient macrophages

**Figure 5:**
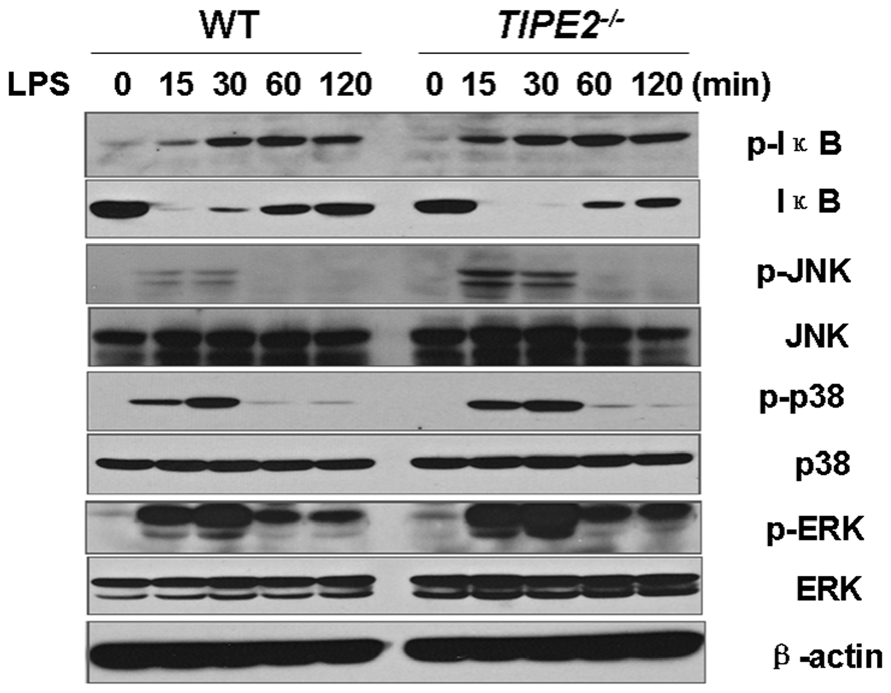
Increased IκBα, JNK and p38 phosphorylation in TIPE2-deficient macrophages. Peritoneal macrophages from WT and *TIPE2^−/−^* mice (n = 4) were incubated with or without LPS (100 ng/mL) for the indicated times. Total cell lysates were examined with antibodies to total or phosphorylated IκBα, JNK1/2, p38 and ERK1/2 by Western blot. β-actin was served as a protein loading control.

As shown in Figure 5, TIPE2 deficiency in macrophages exhibited both enhanced phosphorylation of inhibitor-of-κB (IκB) and degradation of IκB. Compared to WT controls, significant increases in c-Jun N-terminal kinase (JNK) and p38 phosphorylation in TIPE2-deficient macrophages were observed after LPS stimulation, whereas slight increases in p-ERK phosphorylation were noted after LPS stimulation. Taken together, these results suggest that TIPE2 negatively regulates inflammation by inhibiting NF-κB, JNK, and p38 pathways.

## Discussion

In this study, we first address that TIPE2, the tumor necrosis factor-α-induced protein-8 like-2 (TIPE2 or TNFAIP8L2), a newly identified regulator of immune homeostasis, expressed in macrophages constitutes an important component of anti-inflammatory effects by switching arginine metabolism from LPS-induced iNOS to arginase. Using peritoneal macrophages from *TIPE2^−/−^* and WT mice we found that TIPE2-deficiency in macrophages resulted in increases in LPS-induced iNOS protein expression and NO production, and decreases in mRNA levels of arginase I and urea production. However, TIPE2 overexpression in macrophages resulted in decreased protein levels of LPS-induced iNOS and NO, but increased mRNA levels of arginase I and LPS-induced urea production. There were no significant changes in arginase II levels in macrophages with or without TIPE2 expression. Taken together, these findings support our hypothesis that TIPE2 negatively regulates inflammation by switching arginine metabolism from nitric oxide synthase to arginase. This notion was confirmed by the results in LPS-treated *TIPE2^−/−^* mice. We found that *TIPE2^−/−^* mice had higher liver and lung iNOS protein levels and higher plasma NO concentrations, while lower levels of liver arginaseI compared with LPS-treated WT mice.

TIPE2 expression in macrophages can attenuate LPS-induced iNOS expression and NO production, whereas it increases arginase and urea production. These data suggest that in TIPE2 overexpression cells, more L-arg was available to arginase, but not iNOS. On the other hand, deficiency of the *TIPE2* gene in macrophages significantly accelerated LPS-induced iNOS expression and NO production, but led to decreased urea production, suggesting that less L-arg was available to arginase. It has been postulated that NO production from L-arg is involved in the initial host response of inflammatory diseases, while iNOS is considered to be a proinflammatory molecule whose expression is regulated by NF-κB [30]. These findings indicate that TIPE2 can negatively regulate inflammation by downregulating LPS-induced iNOS expression and NO production. Similar phenomena were observed in *TIPE2^−/−^* mice. Higher levels of liver and lung iNOS mRNA and NO concentrations in sera were observed in *TIPE2^−/−^* mice compared to WT controls following LPS challenge.

The higher production of urea in TIPE2 overexpression macrophages may be due to the increased arginase expression following LPS challenge. In TIPE2-deficient macrophages, a lower level of arginaseI was observed compared with WT controls, while the level of iNOS was higher. Therefore, there was a higher level of NO in TIPE2-deficient macrophages with or without LPS-challenge. These findings are consistent with the concept that arginase and NOS compete for a common substrate of intracellular L-arg. NO production can be enhanced by inhibition of arginase [14], [31]–[32]. Switching from NOS to arginase is an important mechanism to limit NO production, which can avoid NO overproduction and negatively regulate inflammation [32].

In this study, increased phosphorylation of JNK, p38, and IκB was observed in TIPE2-deficient macrophages treated with LPS, suggesting that TIPE2 switches NOS to arginase by negatively regulating JNK, NF-κB, and p38 pathways. These results support the notion that TIPE2 is a negative regulator of NF-κB, JNK, and p38 pathways as we have reported recently [1], [12]. Knockout of *TIPE2* would result in the upregulation of phosphorylation of IκB, p38, and JNK, which further resulted in higher levels of iNOS mRNA and protein. iNOS, a macrophage enzyme, induced in inflammation and known to generate O_2_ *^−^* and peroxy nitrite (ONOO*^−^*), is regarded as a kind of proinflammatory cytokine whose expression is regulated by NF-κB [30]. Studies demonstrated that genetic deficiency of iNOS in apoE null mice would cause lighter inflammation reaction and resulted in a decrease in atherosclerosis [33]. It has been reported that LPS stimulates iNOS expression via activation of NF-κB in RAW264.7 cells and that p38 activation is involved in this signaling pathway [34]. The activation of the NF-κB pathway in macrophages leads to more severe inflammatory diseases in mice, possibly by affecting the pro- and anti-inflammatory balance [35]. Here we report that the knockout of *TIPE2* would result in the activation of NF-κB and MAPK signaling pathways, which may contribute to changes in iNOS production as we found in this study. On the contrary, in TIPE2- overexpression cells, both iNOS mRNA and protein levels were decreased.

The levels of arginaseI mRNA were upregulated in LPS treated RAW264.7 macrophages [15], [36]. Because exogenous iNOS and native arginase compete for a common substrate, L-arg, in endothelial cells [37], arginase can represent a molecular mechanism used by macrophages to attenuate NO production and thereby inhibits inflammation reaction [32], [38]–[39]. In this study, we found that the levels of arginaseI mRNA and urea production were decreased in TIPE2-deficient macrophages, while iNOS expression and NO production were increased. On the other hand, TIPE2 overexpression in macrophages could upregulate arginaseI levels, but downregulate iNOS mRNA and protein levels. While the molecular mechanism remains unclear, these data support the hypothesis that TIPE2 may play anti-inflammatory roles by switching arginine metabolism from NOS to arginase. Further studies are required to address this issue.
